# From GRID to gridlock: the relationship between scientific biomedical breakthroughs and HIV/AIDS policy in the US Congress

**DOI:** 10.7448/IAS.16.1.18446

**Published:** 2013-11-27

**Authors:** Matthew B Platt, Manu O Platt

**Affiliations:** 1 Department of Government, Harvard University Cambridge, MA, USA; 2 Wallace H. Coulter Department of Biomedical Engineering, Georgia Institute of Technology and Emory University, Atlanta, GA, USA; 3 Petit Institute of Bioengineering and Biosciences, Georgia Institute of Technology, Atlanta, GA, USA

**Keywords:** agenda setting, PEPFAR, bill sponsorship, bipartisan policy

## Abstract

**Introduction:**

From the travel ban on people living with HIV (PLHIV) to resistance to needle exchange programmes, there are many examples where policy responses to HIV/AIDS in the United States seem divorced from behavioural, public health and sociological evidence. At its root, however, the unknowns about HIV/AIDS lie at biomedical science, and scientific researchers have made tremendous progress over the past 30 years of the epidemic by using antiretroviral therapy to increase the life expectancy of PLHIV almost to the same level as non-infected individuals; but a relationship between biomedical science discoveries and congressional responses to HIV/AIDS has not been studied. Using quantitative approaches, we directly examine the hypothesis that progress in HIV/AIDS biomedical science discoveries would have a correlative relationship with congressional response to HIV/AIDS from 1981 to 2010.

**Methods:**

This study used original data on every bill introduced, hearing held and law passed by the US Congress relating to HIV/AIDS over 30 years (1981–2010). We combined congressional data with the most cited and impactful biomedical research scientific publications over the same time period as a metric of biomedical science breakthroughs. Correlations between congressional policy and biomedical research were then analyzed at the aggregate and individual levels.

**Results:**

Biomedical research advancements helped shape both the level and content of bill sponsorship on HIV/AIDS, but they had no effect on other stages of the legislative process. Examination of the content of bills and biomedical research indicated that science helped transform HIV/AIDS bill sponsorship from a niche concern of liberal Democrats to a bipartisan coalition when Republicans became the majority party. The trade-off for that expansion has been an emphasis on the global epidemic to the detriment of domestic policies and programmes.

**Conclusions:**

Breakthroughs in biomedical science did associate with the number and types of HIV/AIDS bills introduced in Congress, but that relationship did not extend to the passage of laws or to hearings. When science matters, it cannot be separated from political considerations. An important implication of our work has been the depoliticizing role that science can play. Scientific breakthroughs helped to transform HIV/AIDS policy from a niche of liberal Democrats into bipartisan support for the global fight against the disease.

## Introduction

On 30 October 2009, President Barack Obama announced that his administration was ending the travel ban against people living with HIV (PLHIV). Obama described the travel ban as “a decision rooted in fear rather than fact” [1]. In the early days of HIV, when it was called GRID (gay-related immunodeficiency), there was perhaps some basis for the fears that Obama references: pre- and postnatal infant infections [2,3], blood transfusions [4], infected healthcare workers [5] and female heterosexual acquisition [6]. When Congress statutorily enforced the travel ban in 1993 [7], there had been significant advances in understanding transmission [3,8–11], virus detection even in asymptomatic individuals [12] and extending lives with antiretrovirals [13,14]. Despite these important advances, the spectres of Ryan White, Arthur Ashe, Magic Johnson and dentists infecting their patients [10,11] were enough to sustain public fears.

The travel ban is an example of the US Congress responding to public fear and giving it priority over scientific knowledge. This incident is not isolated from other congressional decisions regarding HIV/AIDS. Behavioural, public health and sociological studies have been disregarded: examples include laws disallowing federal funds for needle exchange programs despite their well-documented efficacy for reducing HIV transmission [9,15,16], laws disallowing condom distribution in prisons [17] and the lack of antiretroviral drug availability for the working poor and middle class domestically. Congress has seemed out of step with recommendations from social scientists and public health experts. HIV/AIDS is a biomedical problem with scientific discovery developing new ways to combat it. In this article, we ask: what role has biomedical scientific discovery played in the congressional response to HIV/AIDS?

At its core, this is a study of how and whether science interacts with politics. The science for policy literature suggests that climate change, like HIV/AIDS, is a case where science has been politicized [18]. Jasanoff states that the credibility, trust and validity of science are necessary for scientific progress and public support [19]. Building trust between scientists and the public falls on the scientist, scientific knowledge and the committee advisors who translate these findings for policy purposes [19,20]. In a stylized world, scientific findings are divorced from ideology and partisanship. Debates on climate change took place in a real world where the public lost trust in scientists and detached from their policy positions [19]. As a result, Montpetit argues that climate knowledge is viewed along existing political divisions instead of as objective truth [21].

There are important differences between climate change and HIV/AIDS policy. Firstly, detached HIV/AIDS activists are not possible because the need for treatment ties them to the biomedical community [22]. Secondly, climate change is a long-term, collective disaster, whereas HIV/AIDS (if left untreated) is short term and personal. Biomedical advancements that have extended life to within 10 years of the non-infected life expectancy should reinforce trust in scientists. Along with properly controlled experiments, the rigors of peer review and the clinical evidence of treatments working, presumably without political agenda [23], should create trust between biomedical scientists and the government.

Despite these apparent differences between climate change and HIV/AIDS, their policy fates may be inevitably similar. Guston and colleagues posit that scientific knowledge is not separable from ideology, but has inherent partisanship [24]. A comprehensive study by Hoppe finds a strong consensus that scientific experts and advisors are not partisan and that scientists help to depoliticize hot topics [25]. However, scientific advisors overwhelmingly agreed that scientific expertise plays only a moderate role in policy, and that politics and values are the major influence. Science was used for “uncertainty reduction.” We contribute to this literature by quantitatively testing the hypothesis that biomedical science discoveries in the 30-year fight against HIV/AIDS have a correlative relationship with the congressional response to HIV/AIDS.

## Methods

### Study design

This is a statistical analysis of how congressional policy making – bills introduced, hearings held and laws passed – correlates with the annual number of scientific, biomedical breakthroughs published from 1981 to 2010.

### Data collection

#### HIV/AIDS biomedical scientific literature collection

HIV/AIDS scientific breakthroughs were defined using the most-cited biomedical articles and journals with the highest impact factors (Table 1). Using the Web of Science database (Thomson Reuters) and appropriate search terms, the 500 most-cited articles on HIV/AIDS were identified, and the annual number of articles published from 1981 to 2010 was counted. Articles were ranked by citations per year to avoid bias towards older articles. This measure of scientific knowledge is in line with standard conventions [18]. The cut-off at 500 was arbitrary but sufficient to focus on the most important advances and publications. To confirm reliability, neither the sign nor the significance of results changed when articles were cut off at 1000.

**Table 1:** Biomedical journals with the highest impact factors and top-cited publications for HIV/AIDS research

Impact rank	Journal title	Impact factor
3	*New England Journal of Medicine*	53.5
8	*Nature Genetics*	36.4
9	*Nature*	36.1
11	*Lancet*	33.6
14	*Cell*	32.4
15	*Science*	31.4
18	*Journal of the American Medical Association*	30.0
31	*Nature Immunology*	25.7
32	*Nature Medicine*	25.4
45	*Nature Cell Biology*	19.4
58	*Annals of Internal Medicine*	16.7
63	*PLOS Medicine*	15.6
69	*Journal of Experimental Medicine*	14.8
76	*Journal of Clinical Investigation*	14.2
91	*Genes and Development*	12.9
96	*PLOS Biology*	12.5
124	*Blood*	10.6
151	*Proceedings of the National Academies of Science*	9.8
214	*Cancer Research*	8.2
312	*AIDS*	6.3
384	*Journal of Immunology*	5.7
427	*Journal of Biological Chemistry*	5.3
456	*Journal of Virology*	5.2

As a supplement, a list of important articles was compiled based on our own literature review. This supplemental list allowed the inclusion of influential articles that were published before the HIV/AIDS nomenclature was used or that were not among the top cited but are referenced at international conferences. This included the earliest studies by Montagnier and Gallo from 1981 to 1987, which were nicely summarized [8] during the settlement between these research groups and their governments over initial HIV patents [8,26]. Articles were coded by general subject: transmission, cure, medicine, identification, pathogenesis, women and children, healthcare, homosexual, comorbidities and global epidemic.

#### Congressional policy making

The Congressional Hearing Digital Archive maintained by LexisNexis was searched using “acquired immune deficiency syndrome” to identify hearings related to HIV/AIDS from 1981 to 2010. Our dependent variable for *hearings* was the yearly count of HIV/AIDS hearings in Congress. Data on bill sponsorship and laws passed were also collected. Using the THOMAS database maintained by the US Library of Congress, searches for “AIDS (Disease),” “human immunodeficiency virus” and “HIV/AIDS” were completed. Bills identified from these searches were then cross-referenced with the Congressional Bills Project database. The *bills* variable was the yearly count of HIV/AIDS bills introduced in Congress, and the *laws* variable was the yearly count of HIV/AIDS bills enacted into law. Bills on HIV/AIDS were also coded by subject for consistent comparison to scientific breakthroughs.

#### Control variables

Media coverage [27], partisanship and major HIV/AIDS events in the United States were controlled in the following ways. “Acquired immune deficiency syndrome” was used to search the *New York Times* in LexisNexis, and the *news* variable was the yearly count of those identified articles. We operationalized *partisanship* to take the value of 1 when Democrats held a majority in both houses of Congress, 0 when control was split and −1 when Republicans were the majority [18]. *Major events* was a dummy variable that took the value of 1 for years involving major non-scientific HIV/AIDS events and zero for all others. Major-event years were 1983, when the US Centers for Disease Control and Prevention officially acknowledged AIDS; 1985–1987, when Ryan White was in the press, President Ronald Reagan spoke about AIDS for the first time and azidothymidine was introduced; 1991–1992, when Magic Johnson announced his status of living with HIV; and 1994, when AIDS became the leading killer of men aged 25–44 in the United States.

### Analysis

Statistical software R was used for statistical analysis. Augmented Dickey–Fuller tests showed that none of the five time trends – bills, hearings, laws, scientific breakthroughs or newspaper articles – followed stationary processes. Vector auto-regression (VAR) allowed estimation of the relationship between scientific breakthroughs and congressional response to HIV/AIDS while controlling for reciprocal feedback between media coverage and congressional policy making. The “vars” package was used to run separate models for bills, hearings and laws. The congressional measures and media attention were treated as endogenous variables in a VAR(1) process, and breakthroughs, partisanship and major events were treated as exogenous variables. The breakthrough variable was lagged by one year to allow time for scientific breakthroughs to work through the political system. For example, the following equation was used for bill sponsorship:

$$bills_{t}=\beta_{1}bills_{t-1}+\beta_{2}news_{t-1}+\beta_{3}majorpapers_{t-1}+\beta_{4}democratcontrol_{t}+\beta_{5}majorevents_{t}+\varepsilon_{1t}$$

$$news_{t}=\beta_{6}bills_{t-1}+\beta_{7}news_{t-1}+\beta_{8}majorpapers_{t-1}+\beta_{9}democratcontrol_{t}+\beta_{10}majorevents_{t}+\varepsilon_{2t}$$

#### Analyzing individual-level bill sponsorship

An analysis of bill sponsorship at the individual level supplemented the aggregate. The dependent variable was whether or not a given member of Congress introduced any HIV/AIDS bills in a given year. Standard political science variables of partisanship, ideology, race, gender and institutional position were included with aggregate measures of scientific breakthroughs, media attention and major events. Partisanship was binary: a value of 1 for Democrats and 0 for Republicans. Ideology was measured as the common space score from NOMINATE [28]. These scores range from −1 as most liberal to 1 as most conservative. Institutional position was a binary that took a value of 1 if the member was in the majority party and 0 otherwise. Race and gender were binaries that took values of 1 if member was Black or a woman. Lastly, the total number of bills that a member introduced in a year was included to control for differences in overall legislative activity. The individual-level analysis was conducted using a multilevel logistic regression where effects of ideology, race and partisanship were allowed to vary by year.

## Results

### Annual attention to HIV/AIDS by congressional actions, scientific publications and popular press articles from 1981 to 2010

**Figure 1:**
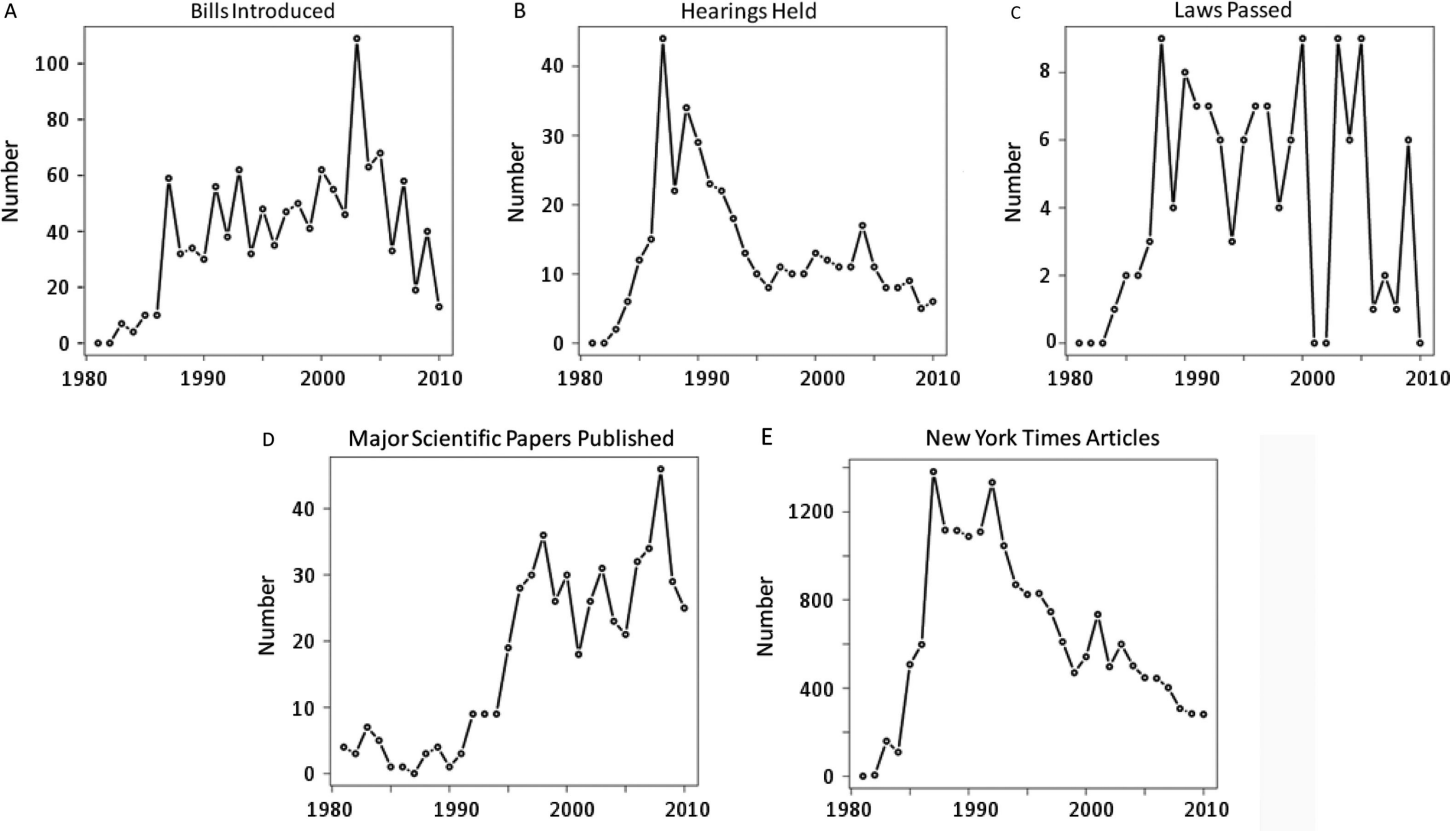
Aggregate data of five major variables used as metrics for attention to HIV/AIDS in the scientific literature, in the US Congress and in the popular press. Databases on scientific publications, congressional hearings, bill sponsorship and *New York Times* articles were searched to compile data on annual numbers of each during the time period of 1981–2010, and they were plotted with the number of each for each year: (A) Bills introduced, (B) hearings held, (C) laws passed, (D) major scientific papers published and (E) *New York Times* articles published.

A data compilation of bills introduced, hearings held, laws passed, major scientific papers published and *New York Times* articles is shown as the number of each by year, from 1981 to 2010 (Figure 1). The number of bills introduced by Congress does not correlate with the number of major scientific papers published each year, but the number of hearings held does look similar to the number of *New York Times* articles published each year.

### Top-cited HIV/AIDS biomedical papers

Looking at the most-cited article from each year between 1986 and 2011, the topics are about identification and testing, viral pathogenesis, treatments and large-scale clinical trials showing efficacy of treatments (Table 2).

**Table 2:** Most cited HIV/AIDS paper of each year from 1986 to 2011 with HIV/AIDS as a keyword and search term

Year	First author	Title	Journal	Times cited
1986	Walker, CM	Lymphocytes-CD8+ can control HIV-infection in vitro by suppressing virus-replication	*Science*	922
1987	Folks, TM	Cytokine-induced expression of HIV-1 in a chronically infected promonocyte cell-line	*Science*	761
1988	Pauwels, R	Rapid and automated tetrazolium-based colorimetric assay for the detection of anti-HIV compounds	*J Virol Meth*	1197
1989	Larder, BA	HIV with reduced sensitivity to zidovudine (AZT) isolated during prolonged therapy	*Science*	1492
1990	Zack, JA	HIV-1 entry into quiescent primary lymphocytes – molecular analysis reveals a labile, latent viral structure	*Cell*	1176
1991	Schreck, R	Reactive oxygen intermediates as apparently widely used messengers in the activation of the NFκB and HIV-1	*EMBO J*	2855
1992	Kohlstaedt, LA	Crystal-structure at 3.5 angstrom resolution of HIV-1 reverse-transcriptase complexed with an inhibitor	*Science*	1464
1993	Pantaleo, G	HIV-infection is active and progressive in lymphoid-tissue during the clinically latent stage of disease	*Nature*	1515
1994	Connor, EM	Reduction of maternal-infant transmission of human-immunodeficiency-virus type-1 with zidovudine treatment	*NEJM*	2112
1995	Ho, DD	Rapid turnover of plasma virions and CD4 lymphocytes in HIV-1 infection	*Nature*	3073
1996	Heid, CA	Real time quantitative PCR	*Genome Res*	3376
1997	Murray, CJL	Alternative projections of mortality and disability by cause 1990–2020: global burden of disease study	*Lancet*	2407
1998	Palella, FJ	Declining morbidity and mortality among patients with advanced human immunodeficiency virus infection	*NEJM*	4942
1999	Schmitz, JE	Control of viremia in simian immunodeficiency virus infection by CD8(+) lymphocytes	*Science*	1372
2000	Paterson, DL	Adherence to protease inhibitor therapy and outcomes in patients with HIV infection	*Ann Intern Med*	1421
2001	Garrus, JE	Tsg101 and the vacuolar protein sorting pathway are essential for HIV-1 budding	*Cell*	721
2002	Sheehy, AM	Isolation of a human gene that inhibits HIV-1 infection and is suppressed by the viral Vif protein	*Nature*	929
2003	Wei, XP	Antibody neutralization and escape by HIV-1	*Nature*	922
2004	Stremlau, M	The cytoplasmic body component TRIM5 alpha restricts HIV-1 infection in Old World monkeys	*Nature*	692
2005	Mattapallil, JJ	Massive infection and loss of memory CD4(+) T cells in multiple tissues during acute SIV infection	*Nature*	590
2006	Lopez, AD	Global and regional burden of disease and risk factors, 2001: systematic analysis of population health data	*Lancet*	1161
2007	Gray, RH	Male circumcision for HIV prevention in men in Rakai, Uganda: a randomised trial	*Lancet*	522
2008	Jones, KE	Global trends in emerging infectious diseases	*Nature*	481
2009	Rerks-Ngarm, S	Vaccination with ALVAC and AIDSVAX to prevent HIV-1 infection in Thailand	*NEJM*	457
2010	Karim, QA	Effectiveness and safety of tenofovir gel, an antiretroviral microbicide, for the prevention of HIV infection in women	*Science*	271
2011	Cohen, MS	Prevention of HIV-1 infection with early antiretroviral therapy	*NEJM*	147

### Scientific breakthroughs correlate with bill sponsorship but not with congressional hearings or laws being passed

Table 3 shows the estimated effects that major HIV/AIDS scientific papers had on sponsoring bills, holding hearings and passing laws. There was a statistically significant relationship only between scientific breakthroughs and bill sponsorship. Years with larger numbers of major papers on HIV/AIDS did not correlate with more hearings or laws passed the following year, which fits findings from the literature for congressional attention [29,30]. Additionally, the number of bills sponsored and laws passed concerning HIV/AIDS increased in response to surges in media attention; the number of hearings held only responded to major events. Trends for hearings and media, illustrated by Figure 1B and 1E, fall in line with major events that occurred between 1985 and 1987 before trailing off after the early 1990s. It seems that congressional hearings and the media move in sync to pay attention to major events relating to HIV/AIDS.

**Table 3:** Members of the US Congress are more likely to introduce HIV/AIDS bills when there have been more major scientific papers published

Variables	Bill sponsorship	Hearings	Laws
**Major papers**	**0.806**	**0.056**	**0.039**
	0.305	0.091	0.034
	*0.014*	*0.546*	*0.261*
**Lagged bills**	**−0.017**		
	0.221		
	*0.938*		
**Lagged hearings**		**0.443**	
		0.293	
		*0.144*	
**Lagged laws**			**−0.192**
			0.196
			*0.338*
**Lagged news**	**0.038**	**0.007**	**0.007**
	0.011	0.007	0.001
	*0.002*	*0.291*	*6.53E-05*
**Democrat control**	**−9.981**	**0.8**	**−1.173**
	5.449	1.987	0.718
	*0.079*	*0.691*	*0.115*
**Major events**	**9.563**	**6.844**	**0.366**
	8.594	3.212	1.173
	*0.277*	*0.043*	*0.758*
**Log likelihood**	**−308.992**	**−268.715**	**−253.516**
***N***	**29**	**29**	**29**

Effects that major scientific papers about HIV/AIDS had on sponsoring bills, holding hearings and passing laws were estimated as a VAR(1) process. The coefficients from that estimation are presented in Table 3 in boldface, along with the standard errors in roman type and the *p*-values in italics. When there are more major scientific papers published on HIV/AIDS and/or there are more news articles about HIV/AIDS, then there should be more HIV/AIDS bills introduced in Congress the following year.

### Merging political science and biomedical science to explain HIV/AIDS bill sponsorship

In the aggregate, biomedical science has played a role in getting HIV/AIDS bills sponsored but not passed into law. This also held at the individual level (Table 4).

**Table 4:** Independent variables’ effect on the probability of an individual US Congress member introducing HIV/AIDS legislation

Variables	Change in probability	95% confidence interval	House results	Senate results
*Major papers	0.7%	[0.5%, 0.9%]	0.6% [0.4, 0.9]*	1.0% [0.5, 1.6]*
*Ideology	−2%	[−2.8%, −1.2%]	−2.2% [−3.4, −1.3]*	−1.9% [−4.1, −0.4]*
*Democrat	−2.5%	[−3.7%, −1.6%]	−2.8% [−4.3, −1.7]*	−2.2% [−5.2, −0.03]*
*Female	1.1%	[0.1%, 2.3%]	2.3% [0.9, 4.3]*	−0.7% [−2.6, 1.4]
Black	0.1%	[−1.1%, 1.6%]	0.5% [−0.8, 2.0]	7.9% [−4.7, 64]
*Majority	2.1%	[1.2%, 3.2%]	3.2% [1.9, 4.8]*	1.0% [−0.5, 2.9]
*Total bills	2.3%	[1.6%, 3.0%]	1.8% [1.2, 2.6]*	2.5% [1.4, 4.1]*
*News	2%	[1.4%, 2.9%]	1.4% [0.8, 2.2]*	3.4% [1.6, 5.6]*
Major events	1.1%	[−0.02%, 2.3%]	1.0% [−0.4, 2.5]	1.4% [−1.1, 4.4]

Predicted change in the probability of the variable (given in each row) to effect change in an individual Congress member to introduce an HIV/AIDS bill was derived from multilevel logistic regression. 95% confidence intervals are shown as well. If the confidence interval does not contain zero, then the predicted change is statistically significant.

* Denotes statistical significance. The first two columns are for the full data set that combines the House and Senate. The last two columns show separate results for House members and Senate members, respectively.

**Figure 2:**
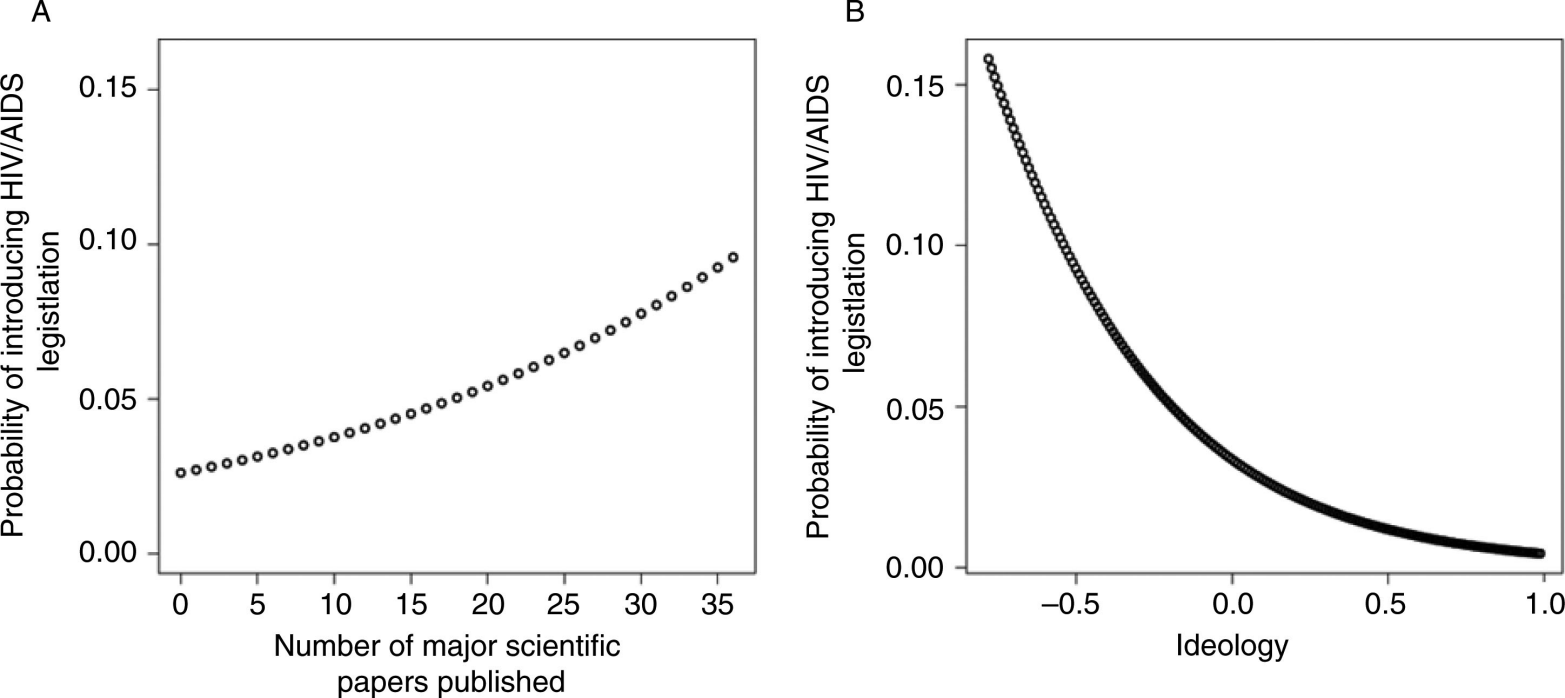
Biomedical breakthroughs and liberal ideology increase sponsorship of HIV/AIDS bills. Predicted probabilities derived from multilevel logistic regression on individual-level bill sponsorship of HIV/AIDS legislation: (A) The probability of introducing a bill on HIV/AIDS as the number of scientific breakthroughs increases; and (B) the probability of introducing a bill on HIV/AIDS as ideology moves from liberal to conservative.

Figure 2A illustrates how the probability of bill sponsorship increased when the number of major scientific papers published each year increased. Given the relatively low probability that a member of Congress would introduce HIV/AIDS bills, the threefold change from 3 to 9% (over a range from 0 to 35 papers) could be considered substantial.

Ideology also had dramatic effects on introducing HIV/AIDS legislation (Figure 2B). Conservative members of Congress were far less likely to introduce bills on HIV/AIDS issues. Women were 1% more likely to sponsor legislation. Being in the majority party has consistently been related to increases in bill sponsorship [31,32] and conveys a two-percentage-point increase for introducing HIV/AIDS bills. Republicans being two percentage points more likely to introduce HIV/AIDS bills initially seems counterintuitive. The correct interpretation is that liberal and moderate Republicans are more active than conservative Democrats. For example, there are eleven members of Congress (MCs) who fall within the ideologically moderate range of −0.1 and 0.1 (conservative Democrats and liberal Republicans) and who have introduced HIV/AIDS bills. Of those 11 MCs, only two are Democrats. We need to look closer at the content of the breakthroughs and the bills to build on the correlations revealed by the aggregate- and individual-level analysis.

### Congressional attention to HIV/AIDS segregated by topics

**Figure 3:**
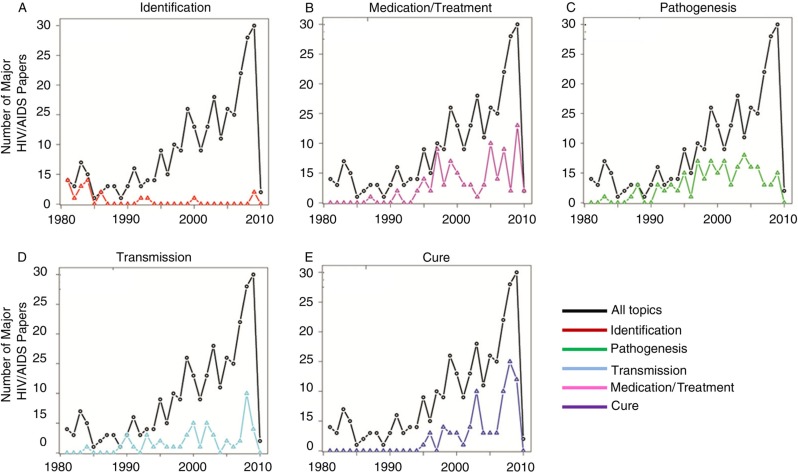
Major scientific breakthrough publications categorized by topic for each year compared to the total number of HIV/AIDS articles published. The top 500 most-cited papers were categorized according to (A) identification, (B) pathogenesis, (C) transmission, (D) medication and treatment and (E) cure. Total numbers of papers published per year were calculated and plotted.

A brief summary of the most-cited papers in high-impact journals reveals a coherent story about how scientific breakthroughs compared with congressional attention. In the 1980s, HIV/AIDS scientific breakthroughs centred on Montagnier, Barre-Sinnousi and Gallo isolating and culturing HIV, developing antibodies and research reagents for molecular and cellular studies and understanding the mechanisms of infection and transmission [33–38]. Identification dominated major papers in the 1980s (Figure 3A).

Scientific advances in the 1990s shifted towards medicine and treatment (Figure 3B), as well as pathogenesis and transmission studies (including comorbidities and the side effects of antiretrovirals) (Figure 3C and 3D). Key biomedical findings during this period include sensitive, quantitative detection of viral RNA [12,39,40] and crystallization of HIV-1 reverse transcriptase [41]. This accelerated the development of pharmaceutical inhibitors targeting this key enzyme. Today, these drugs have saved 3 million years of life [42,43] and are in 100% of HIV medication cocktails.

The earliest scientific discussions on curing the disease (Figure 3E), in a real sense, began in 1996 after researchers discovered people who had a CCR5 mutated co-receptor that inhibited infection and slowed disease progression to AIDS without the use of antiretrovirals [44–47]. Removing long-term, undetectable-viral-load patients from antiretrovirals saw rebounded viral loads, plummeting CD4 cells and the realization that patients were not cured but that viral reservoirs were established in latent infected cells [48,49]. Scientists then developed fusion and integrase inhibitors to block the entry and establishment of viral reservoirs in cells [50]. HIV vaccine development suffered a setback when a major trial failed in 2003 [51,52]. Lastly, there was a surge in papers on transmission and on medication and treatment from 2000 to 2010, once biomedical scientists determined that successful viral suppression with highly active antiretroviral therapy (HAART) also lowered the risk of transmission to non-infected individuals [53,54].

**Figure 4:**
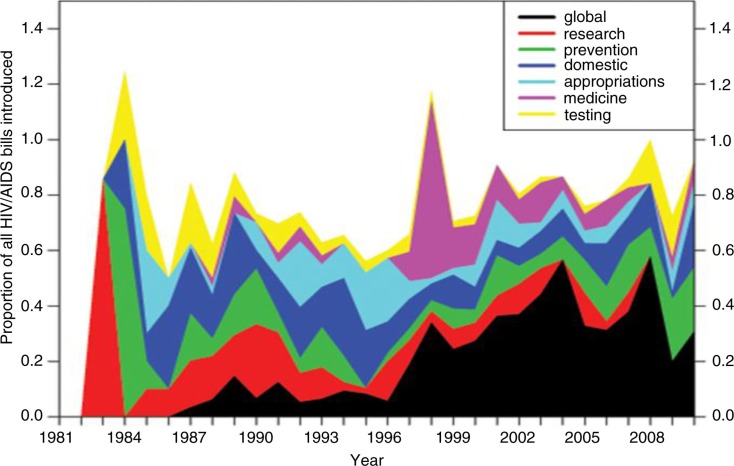
Changes in the composition of the HIV/AIDS congressional agenda over 30 years of the epidemic. All HIV/AIDS bills introduced in Congress from 1981 to 2010 were coded according to subjects and tabulated.

All bills introduced and major papers on HIV/AIDS were coded according to predetermined subjects. Figure 4 shows how the legislative agenda for HIV/AIDS changed over time. During the 1980s, bills were introduced to fund research, adopt guidelines and programmes for testing, establish social programmes for PLWHA and help with prevention. By the 1990s, many bills were about maintaining, appropriating and, in some instances, slashing funding for these established programmes. The largest correlative peak between the science of and the congressional response to HIV/AIDS occurred in 1997 (Figures 3 and 4) with the discovery and implementation of HAART, the three-drug cocktail, to effectively suppress viral levels [55–58], leading to the first-ever drop in AIDS deaths [59,60]. Concurrently, the global epidemic began to dominate the legislative agenda, accounting for 50% of all HIV/AIDS bills introduced in this time frame (Figure 4). This may have been due to major findings from large clinical trials conducted at foreign research sites, which helped illuminate the global epidemic to the United States.

### Biomedical research breakthroughs foster bipartisan HIV/AIDS policy

**Figure 5:**
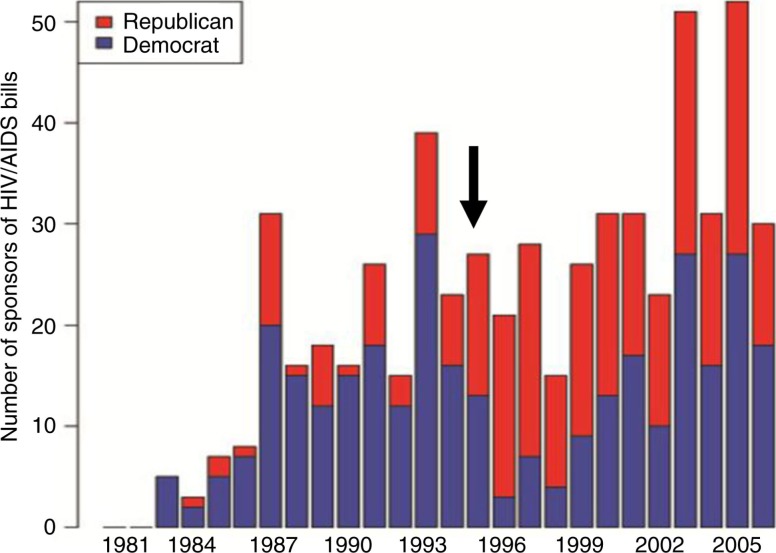
Increased sponsorship of HIV/AIDS bills by Republicans over time. Individual congress members’ introductions of HIV/AIDS bills were tabulated based on party affiliation. Arrow indicates the 1994 midterm elections, which led to a shift from mostly Democrat to more bipartisan contributions.

The last piece of the story looks more closely at who sponsors HIV/AIDS bills. From 1983 to 1994, Democrats dominated the introduction of bills in response to the HIV/AIDS crisis, but when Republicans were swept into power in the 1994 midterm elections, there was a stark partisan shift in who introduced HIV/AIDS bills, and it became a bipartisan issue. Republicans moved away from punitive “culture war” policies of the 1990s, and in the early 2000s, powered by the “compassionate conservatism” of President George W. Bush, joined efforts to fight HIV/AIDS globally, including the passage of the President's Emergency Plan for AIDS Relief (PEPFAR), resulting in a decade of bipartisan HIV/AIDS bill sponsorship (Figure 5).

Table 5 provides more insight into the politics of congressional attention to HIV/AIDS. Half of the top 10 sponsors of HIV/AIDS bills represented California. This was the epicentre of the disease in its early years, so it makes sense that these MCs would feel a greater need to respond to the crisis. Ted Kennedy's senate career was defined by a longstanding commitment to healthcare issues, so his inclusion at the top of this list is not surprising. Conservative Republican William Dannemeyer's bills are all about required HIV testing and subsequent prohibitions of PLWHA from various public health occupations. All of Henry Hyde's and James Walsh's bills are related to their positions as committee and/or subcommittee chairmen. A closer look at who sponsors HIV/AIDS bills shows that institutional position, potential constituency pressures, partisanship and ideology – in other words, politics – are part of how biomedical breakthroughs are translated into the congressional response to HIV/AIDS.

**Table 5:** Liberal California Democrats are the most active regarding HIV/AIDS in Congress

Name	No. of HIV bills	Ideology	Party
Henry Waxman	35	−0.514	Democrat
Ted Kennedy	30	−0.479	Democrat
Barbara Lee	17	−0.743	Democrat
Maxine Waters	16	−0.713	Democrat
Henry Hyde	13	0.313	Republican
William Dannemeyer	13	0.689	Republican
Nancy Pelosi	12	−0.551	Democrat
Arlen Specter	11	0.070	Republican
James McDermott	11	−0.717	Democrat
James Walsh	11	0.200	Republican

This table lists the 10 members of Congress (MCs) who introduced the highest number of HIV/AIDS bills during the period of our study. The columns show the total number of HIV/AIDS bills introduced, the ideology of the MC as measured by common space NOMINATE scores (−1 is the most liberal and +1 is the most conservative) and the party affiliation.

## Discussion

In the aggregate and at the individual level, science had a real – although limited – role to play in congressional policy making concerning HIV/AIDS. For HIV/AIDS, major scientific papers correlated with bill sponsorship by members of Congress. Scientific breakthroughs did not impact legislative hearings or the passage of bills into law. Sponsoring bills requires lower thresholds of attention, and given this, it makes sense that biomedical scientific advances matter for bill sponsorship rather than hearings or laws.

We interpret these results through the lens of the agenda-setting literature in political science. Accordingly, policy entrepreneurs define or redefine issues for appeal to broader audiences, and then those audiences help to break established policy monopolies that are keeping new issues off the agenda [61,27,29,62–65]. Bill sponsorship fits into that entrepreneurial role [66]. Table 5 illustrates that MCs took on an entrepreneurial role for policy, electoral and institutional reasons [31,67]. Increasing numbers of scientific breakthroughs provide more opportunities for entrepreneurs, leading to more HIV/AIDS bills being introduced. As seen in Figures 3 and 4, early breakthroughs were essential to defining HIV/AIDS as a new national health crisis. Due to the limits of biomedical science at the time, policy solutions were education, prevention and research funding. Later breakthroughs in medical treatments provided for new policy solutions such as subsidized access to treatment.

Kingdon [62] argues that a well-defined problem and plausible policy solution are not enough to move items onto Congress's formal agenda. It is important to note that our study concerns only the biomedical science portion of the HIV/AIDS landscape. Activism around the disease, public health (as opposed to strictly biomedical) research and other forms of advocacy are essential for providing a full picture of how and why Congress paid attention to HIV/AIDS. It is entirely possible that these other aspects fill in the gaps that biomedical research leaves regarding hearings and passing laws.

Figures 3, 4 and 5 provide a picture of how ideology, partisanship and science shaped congressional responses. For its first 15 years, HIV and AIDS were viewed, together, primarily as the disease of drug addicts and gay White men, hence the earlier name of GRID. With that, it was relegated to being a niche liberal issue. The Democrats who controlled Congress responded with bills for social programmes for prevention, testing and research, but GRID met the gridlock of Congress and only as many as eight bills passed per year out of the 50–100 that were introduced (Figure 1). During this time, scientists worked to isolate and identify this virus [33–38]. In the mid-1990s, Republicans won control of Congress and scientific breakthroughs shifted towards more effective medical treatments. Domestically, the newly infected shifted from gay White men more to those who were Black, Latino, poor, deviant and, in the minds of some, undeserving of help [68–70]. By 2000, Blacks and Latinos had surpassed Whites in AIDS-related deaths [71]. Globally, there was an opportunity for Republicans to seize policy initiatives on HIV/AIDS and build their brand of compassionate conservatism by using scientific advances in treatments for HIV/AIDS to provide medicines for poor countries through PEPFAR.

It is interesting to postulate what role the latest biomedical breakthroughs will play in congressional attention and domestic policy going forward, with the success of the CAPRISA (Centre for the AIDS Programme of Research in South Africa) trial, in which a vaginal microbicide was shown to reduce transmission to women through heterosexual contact [72], and the iPREX (Pre-Exposure Prophylaxis Initiative) trial that showed the efficacy of pre-exposure prophylaxis (PrEP) with antiretrovirals in preventing new HIV infections among men who have sex with men (MSM) [73]. CAPRISA provides a means for women to protect themselves, but iPREX demonstrated efficacy for the high-risk (yet still culturally stigmatized) group of MSM, a group that also does not find favour with religious and political conservatives. Whether one will find more favour compared to the other in the eyes of Congress, to receive federal funds for treatment or public health distribution, remains to be seen. Perhaps Guston was right and the science cannot be separated from the politics, or at least not in the way that biomedical scientists believe it should be. Nor can it be separated from advocates and public health issues to maximize congressional response. Non-scientific methods must be involved as well, and while science is helpful, it must be supplemented “with the analysis of those aspects of the human condition that science cannot easily illuminate” [74].

## Conclusions

We have shown that breakthroughs in biomedical research did associate with the number and types of HIV/AIDS bills introduced in Congress, but that relationship did not extend to passage of laws or to congressional hearings. We began by asking whether science could shape policy without itself being shaped by politics. This study makes three contributions to the literature around that question. Firstly, our findings are slightly counterintuitive because we show that science can matter for policy making. The agenda-setting literature suggests that complex information, like biomedical research, would not impact bill sponsorship. Secondly, we provide a more direct quantitative test of the hypothesized relationship between science and policy. Thirdly, we provide additional nuance to the science-for-policy debate. We have argued that biomedical breakthroughs created opportunities for policy entrepreneurs – that, in effect, science opened a way for politics. However, the ultimate consequence has been a depoliticization of HIV/AIDS policy at the congressional level. Scientific breakthroughs helped to transform HIV/AIDS policy from a niche of liberal Democrats to bipartisan support to fight the disease globally. In that regard, these findings bring us closer to Guston's position. Science matters when it suits the politics.

## References

[1] Obama B, Peters G, Woolley JT (2009). Remarks on signing the Ryan White HIV/AIDS Treatment Extension Act of 2009. The American presidency project.

[2] Centers for Disease Control (CDC) (1982). Unexplained immunodeficiency and opportunistic infections in infants – New York, New Jersey, California. MMWR Morb Mortal Wkly Rep.

[3] Ziegler JB, Cooper DA, Johnson RO, Gold J (1985). Postnatal transmission of AIDS-associated retrovirus from mother to infant. Lancet.

[4] Centers for Disease Control (CDC) (1982). Possible transfusion-associated acquired immune deficiency syndrome (AIDS) – California. MMWR Morb Mortal Wkly Rep.

[5] Centers for Disease Control (CDC) (1983). Acquired immunodeficiency syndrome (AIDS): precautions for health-care workers and allied professionals. MMWR Morb Mortal Wkly Rep.

[6] Centers for Disease Control (CDC) (1983). Immunodeficiency among female sexual partners of males with acquired immune deficiency syndrome (AIDS) – New York. MMWR Morb Mortal Wkly Rep.

[7] Preston J (2009). Obama lifts a ban on entry into U.S. by HIV-positive people.

[8] Gallo RC, Montagnier L (1987). The chronology of AIDS research. Nature.

[9] (1988). Intravenous drug abuse speeds the spread of AIDS. Netw Res Triangle Park N C.

[10] Centers for Disease Control (CDC) (1990). Possible transmission of human immunodeficiency virus to a patient during an invasive dental procedure. MMWR Morb Mortal Wkly Rep.

[11] Centers for Disease Control (CDC) (1991). Transmission of HIV-1 infection during an invasive dental procedure – United States. CDR (Lond Engl Wkly).

[12] Piatak M, Saag MS, Yang LC, Clark SJ, Kappes JC, Luk KC (1993). High levels of HIV-1 in plasma during all stages of infection determined by competitive PCR. Science.

[13] van Leeuwen R, Lange JM, Hussey EK, Donn KH, Hall ST, Harker AJ (1992). The safety and pharmacokinetics of a reverse transcriptase inhibitor, 3TC, in patients with HIV infection: a phase I study. AIDS.

[14] Fischl MA, Richman DD, Grieco MH, Gottlieb MS, Volberding PA, Laskin OL (1987). The efficacy of azidothymidine (AZT) in the treatment of patients with AIDS and AIDS-related complex. A double-blind, placebo-controlled trial. N Engl J Med.

[15] Lurie P, Drucker E (1997). An opportunity lost: HIV infections associated with lack of a national needle-exchange programme in the USA. Lancet.

[16] Roberts J (1995). Needle exchanges reduce HIV infection in US. BMJ.

[17] Centers for Disease Control (CDC) (2006). HIV transmission among male inmates in a state prison system – Georgia, 1992–2005. MMWR Morb Mortal Wkly Rep.

[18] Liu X, Lindquist E, Vedlitz A (2011). Explaining media and congressional attention to global climate change, 1969–2005: an empirical test of agenda setting theory. Polit Res Q.

[19] Jasanoff S (2010). Science and society. Testing time for climate science. Science.

[20] Jasanoff S (1990). The fifth branch: science advisers as policymakers.

[21] Montpetit E (2011). Scientific credibility, disagreement, and error costs in 17 biotechnology policy subsystems. Pol Stud J.

[22] Epstein S (1996). Impure science: AIDS, activism, and the politics of knowledge.

[23] Alberts B (2008). A scientific approach to policy. Science.

[24] Guston DH, Sarewitz D, Miller C (2009). Scientists not immune to partisanship. Science.

[25] Hoppe R (2009). Scientific advice and public policy: expert advisers’ and policymakers’ discourses on boundary work. Poiesis Praxis.

[26] Palca J (1987). Settlement on AIDS finally reached between US and Pasteur. Nature.

[27] Cobb R, Ross J-K, Ross MH (1976). Agenda building as a comparative political process. Am Polit Sci Rev.

[28] Poole KT (1998). Recovering a basic space from a set of issue scales. Am J Polit Sci.

[29] Baumgartner FR, Jones BD (1993). Agendas and instability in American politics.

[30] Jones BD, Baumgartner FR (2005). The politics of attention: how government prioritizes problems.

[31] Schiller WJ (1995). Senators as political entrepreneurs: using bill sponsorship to shape legislative agendas. Am J Polit Sci.

[32] Cox GW, Terry WC (2008). Legislative productivity in the 93rd–105th Congresses. Legis Stud Q.

[33] Gallo RC, Salahuddin SZ, Popovic M, Shearer GM, Kaplan M, Haynes BF (1984). Frequent detection and isolation of cytopathic retroviruses (HTLV-III) from patients with AIDS and at risk for AIDS. Science.

[34] Popovic M, Sarngadharan MG, Read E, Gallo RC (1984). Detection, isolation, and continuous production of cytopathic retroviruses (HTLV-III) from patients with AIDS and pre-AIDS. Science.

[35] Sarngadharan MG, Popovic M, Bruch L, Schupbach J, Gallo RC (1984). Antibodies reactive with human T-lymphotropic retroviruses (HTLV-III) in the serum of patients with AIDS. Science.

[36] Schupbach J, Popovic M, Gilden RV, Gonda MA, Sarngadharan MG, Gallo RC (1984). Serological analysis of a subgroup of human T-lymphotropic retroviruses (HTLV-III) associated with AIDS. Science.

[37] Barre-Sinoussi F, Chermann JC, Rey F, Nugeyre MT, Chamaret S, Gruest J (1983). Isolation of a T-lymphotropic retrovirus from a patient at risk for acquired immune deficiency syndrome (AIDS). Science.

[38] Kalyanaraman VS, Cabradilla CD, Getchell JP, Narayanan R, Braff EH, Chermann JC (1984). Antibodies to the core protein of lymphadenopathy-associated virus (LAV) in patients with AIDS. Science.

[39] Heid CA, Stevens J, Livak KJ, Williams PM (1996). Real time quantitative PCR. Genome Res.

[40] Ogg GS, Jin X, Bonhoeffer S, Dunbar PR, Nowak MA, Monard S (1998). Quantitation of HIV-1-specific cytotoxic T lymphocytes and plasma load of viral RNA. Science.

[41] Kohlstaedt LA, Wang J, Friedman JM, Rice PA, Steitz TA (1992). Crystal structure at 3.5 A resolution of HIV-1 reverse transcriptase complexed with an inhibitor. Science.

[42] De Clercq E (2009). Anti-HIV drugs: 25 compounds approved within 25 years after the discovery of HIV. Int J Antimicrob Agents.

[43] Walensky RP, Paltiel AD, Losina E, Mercincavage LM, Schackman BR, Sax PE (2006). The survival benefits of AIDS treatment in the United States. J Infect Dis.

[44] Samson M, Libert F, Doranz BJ, Rucker J, Liesnard C, Farber CM (1996). Resistance to HIV-1 infection in caucasian individuals bearing mutant alleles of the CCR-5 chemokine receptor gene. Nature.

[45] Rowland-Jones SL, McMichael A (1995). Immune responses in HIV-exposed seronegatives: have they repelled the virus?. Curr Opin Immunol.

[46] Liu R, Paxton WA, Choe S, Ceradini D, Martin SR, Horuk R (1996). Homozygous defect in HIV-1 coreceptor accounts for resistance of some multiply-exposed individuals to HIV-1 infection. Cell.

[47] Stranford SA, Skurnick J, Louria D, Osmond D, Chang SY, Sninsky J (1999). Lack of infection in HIV-exposed individuals is associated with a strong CD8(+) cell noncytotoxic anti-HIV response. Proc Natl Acad Sci USA.

[48] Chun TW, Carruth L, Finzi D, Shen X, DiGiuseppe JA, Taylor H (1997). Quantification of latent tissue reservoirs and total body viral load in HIV-1 infection. Nature.

[49] Chun TW, Stuyver L, Mizell SB, Ehler LA, Mican JA, Baseler M (1997). Presence of an inducible HIV-1 latent reservoir during highly active antiretroviral therapy. Proc Natl Acad Sci USA.

[50] Lalezari JP, Henry K, O'Hearn M, Montaner JS, Piliero PJ, Trottier B (2003). Enfuvirtide, an HIV-1 fusion inhibitor, for drug-resistant HIV infection in North and South America. N Engl J Med.

[51] Buchbinder SP, Mehrotra DV, Duerr A, Fitzgerald DW, Mogg R, Li D (2008). Efficacy assessment of a cell-mediated immunity HIV-1 vaccine (the Step Study): a double-blind, randomised, placebo-controlled, test-of-concept trial. Lancet.

[52] McCarthy M (2003). HIV vaccine fails in phase 3 trial. Lancet.

[53] Pitcher CJ, Quittner C, Peterson DM, Connors M, Koup RA, Maino VC (1999). HIV-1-specific CD4+ T cells are detectable in most individuals with active HIV-1 infection, but decline with prolonged viral suppression. Nat Med.

[54] Furtado MR, Callaway DS, Phair JP, Kunstman KJ, Stanton JL, Macken CA (1999). Persistence of HIV-1 transcription in peripheral-blood mononuclear cells in patients receiving potent antiretroviral therapy. N Engl J Med.

[55] Mauss S, Adams O, Willers R, Jablonowski H (1996). Combination therapy with ZDV + DDI versus ZDV + DDC in patients with progression of HIV-infection under treatment with ZDV. J Acquir Immune Defic Syndr Hum Retrovirol.

[56] Autran B, Carcelain G, Li TS, Blanc C, Mathez D, Tubiana R (1997). Positive effects of combined antiretroviral therapy on CD4+ T cell homeostasis and function in advanced HIV disease. Science.

[57] Gulick RM, Mellors JW, Havlir D, Eron JJ, Gonzalez C, McMahon D (1997). Treatment with indinavir, zidovudine, and lamivudine in adults with human immunodeficiency virus infection and prior antiretroviral therapy. N Engl J Med.

[58] Hammer SM, Squires KE, Hughes MD, Grimes JM, Demeter LM, Currier JS (1997). A controlled trial of two nucleoside analogues plus indinavir in persons with human immunodeficiency virus infection and CD4 cell counts of 200 per cubic millimeter or less. AIDS Clinical Trials Group 320 Study Team. N Engl J Med.

[59] Hogg RS, O'Shaughnessy MV, Gataric N, Yip B, Craib K, Schechter MT (1997). Decline in deaths from AIDS due to new antiretrovirals. Lancet.

[60] Palella FJ, Delaney KM, Moorman AC, Loveless MO, Fuhrer J, Satten GA (1998). Declining morbidity and mortality among patients with advanced human immunodeficiency virus infection. HIV Outpatient Study Investigators. N Engl J Med.

[61] Cobb RW, Elder CD (1971). Politics of agenda-building – alternative perspective for modern democratic theory. J Polit.

[62] Kingdon JW (1995). Agendas, alternatives, and public policies.

[63] Baumgartner FR, Jones BD, MacLeod MC (2000). The evolution of legislative jurisdictions. J Polit.

[64] Schattschneider EE (1960). The semisovereign people: a realist's view of democracy in America.

[65] Sheingate AD (2006). Structure and opportunity: committee jurisdiction and issue attention in Congress. Am J Polit Sci.

[66] Wawro GJ (2000). Legislative entrepreneurship in the U.S. House of Representatives.

[67] Mayhew D (1974). Congress: the electoral connection.

[68] Armstrong EM, Carpenter DP, Hojnacki M (2006). Whose deaths matter? mortality, advocacy, and attention to disease in the mass media. J Health Polit Pol Law.

[69] Gilens M (1996). “Race coding” and white opposition to welfare. Am Polit Sci Rev.

[70] Cohen CJ (1999). The boundaries of blackness: AIDS and the breakdown of black politics.

[71] Centers for Disease Control (CDC) (2000). HIV/AIDS among racial/ethnic minority men who have sex with men – United States, 1989–1998. MMWR Morb Mortal Wkly Rep.

[72] Abdool Karim Q, Abdool Karim SS, Frohlich JA, Grobler AC, Baxter C, Mansoor LE (2010). Effectiveness and safety of tenofovir gel, an antiretroviral microbicide, for the prevention of HIV infection in women. Science.

[73] Grant RM, Lama JR, Anderson PL, McMahan V, Liu AY, Vargas L (2010). Preexposure chemoprophylaxis for HIV prevention in men who have sex with men. N Engl J Med.

[74] Jasanoff S (2007). Technologies of humility. Nature.

